# Supplementary material for: Point-of-Care Ultrasound (POCUS) in Pediatric Practice in Poland: Perceptions, Competency, and Barriers to Implementation—A National Cross-Sectional Survey
Source: Healthcare (Basel). 2025 Aug 5;13(15):1910. doi: 10.3390/healthcare13151910 (PMC12345954; doi:10.3390/healthcare13151910)
Supplement: Supplementary file 1 [file healthcare-13-01910-s001.zip › healthcare-3716485-supplementary.pdf]

Supplementary Table 1. Advantages of POCUS according to the respondents' opinions, open- field question.

No.	Category	Quote
1	Reducing unnecessary referrals	Respondent M99 *"The ability to perform POCUS examinations in primary care offices will shorten waiting times for diagnostic services and outpatient specialist clinics."*
		Respondent M105 *"Reduction in the number of laboratory and imaging tests performed; fewer patients referred to specialist outpatient clinics."*
2	Cost savings	Respondent M19 *"Reduction in diagnostic costs and medication use, such as antibiotics; consistent and easy patient monitoring; increased physician autonomy."*
		Respondent M165 *"Public cost savings – faster and more accurate diagnoses lead to more effective and timely treatment, saving both time (for doctors and parents) and money (e.g., social insurance costs for parental sick leave, public healthcare expenses for medications and staff salaries)."*
3	Differentiating infections	Respondent *M83 "Differentiation of acute infectious conditions and reduction of unnecessary antibiotic treatments."*
		Respondent M152 *"Invaluable support in the diagnosis of pneumonia, bronchitis, and bronchiolitis, especially in the youngest and non-cooperative patients."*
4	Antibiotic stewardship	Respondent M24*"Reduction of unnecessary antibiotic therapy (e.g., in bronchiolitis in infants); faster implementation of appropriate treatment when constipation is confirmed; direct referral to the appropriate department (e.g., deciding whether to refer to pediatrics or pediatric surgery in cases of abdominal pain)."*
		Respondent M30 *"Decreases the use of empirical antibiotics – better targeted treatment".*
5	Caregiver communication and patient comfort	Respondent M67 *"Greater parental trust, increased confidence in the diagnosis, and potentially better compliance."*
		Respondent M207 *"Reduced stress for patients."*

Note: Categories are not ordered by frequency.

Supplementary Table 2. Barriers to performing POCUS according to the respondents' opinions, expressed through an open-ended question.

No.	Category	Quote
1	Lack of time	Respondent P2 *"Too many patients or too little time per patient in primary care."*
		Respondent P44 *"Lack of time for physicians to pursue training in this area."*
2	Resistance to change	Respondent P165 *"Generational barrier and fear of new technologies."*
		Respondent P187 *"Lack of trust—especially among senior physicians—in the information obtained through POCUS, and the fact that these results are often not taken into account during diagnosis and clinical decision-making."*
3	Cultural barriers	Respondent P53 *"Reluctance among senior pediatricians – 'we didn't have this before, and we managed just fine.'"*
		Respondent P156 *"The belief among practicing pediatricians that ultrasound is a procedure reserved exclusively for radiologists. Especially when no radiologist is on duty due to administrative decisions, based on the assumption that pediatricians can perform ultrasound themselves."*
4	Lack of motivation or leadership support	Respondent P117 *„Lack of engagement and willingness to learn among part of the medical staff."*
		Respondent P152 *"Lack of support from medical authorities."*
5	Financial barriers	Respondent P43 *„Cost of training"*
		Respondent P24 *"High cost of training and its limited availability (in terms of scheduling and location)."*

Note: Categories are not ordered by frequency.

Supplementary Table 3. Strategies to Improve POCUS Implementation (an open-ended question).

	Category	Quote
1	Funding and institutional support	*Respondent Q2:"Education, training, guidelines, and financial incentives for additional skills provided by management and the National Health Fund (NFZ)."*
		Respondent Q3: "Training organized, for example, by Centrum Medyczne Kształcenia Podyplomowego (Center of Postgraduate Medical Education, CMKP) during specialist training, and affordable courses for medical specialists organized by the Regional Medical Chamber (OIL). Changes in work organization – for instance, scheduled breaks in the work plan for performing ultrasound exams (including time needed to move between rooms, set up the equipment, and perform the examination)."
2	Integration into specialization curriculum	*Respondent Q56: "Include ultrasound training in residency, not just as optional courses".*
		RespondentQ85 ""Greater availability of courses in hospitals during the current stage of specialist training."
		Q198"Incorporation of POCUS skills into the pediatric specialization curriculum. Requiring the availability of POCUS equipment in pediatric wards as one of the criteria for granting accreditation to conduct pediatric specialization training."
3	Access to equipment	*Q121"Availability of equipment in outpatient settings and sufficient time allocated per visit to carry out the examination."*
		RespondentQ148" purchase more portable, small-sized ultrasound devices"
4	Time for visit	*RespondentQ1 "having more time for each patient and better triage"*
5	Professional society support	*RespondentQ67 “"Spreading knowledge through forums, conferences, and professional journals."*
		RespondentQ179"Support from scientific societies"

Note: Categories are not ordered by frequency.

Supplementary Table 4. Comparison of Pediatric POCUS Course Offerings in Selected High-Income Countries

Country	Main Provider(s)	Course Duration	Approximate Fee (EUR)
**Poland**	Roztoczaska USG School, EBA, Eduson, Siemens Academy	1-5 days	450-900
**Germany**	Sonokolleg, St. Marien, Magdeburg Univ. Hosp., IPUN, EUSEM	1-3 days or part of longer programs, institutional support	400-1300
**Italy**	Bambino Ges (Tor Vergata), Meyer Hospital	2-3 days or part of longer programs, institutional support	300-1000
**Canada**	CanPoCUS, SickKids Toronto	2-3 days or integrated in PEM fellowships	500-1000
**USA**	Stanford, Baylor, Children's Colorado	2-3 days or integrated in PEM fellowships	600-1100

Note: The data are approximate and based on publicly available information from training providers. In Canada and the USA, pediatric POCUS training is often embedded in structured pediatric emergency medicine (PEM) curricula and supported by institutional programs or fellowships. In contrast, in Poland, training is typically fee-based and individually initiated.

Supplementary File

POCUS in Pediatric Practice in Poland: A Nationwide Survey

English translation of the questionnaire used in the study (original language: Polish)

Dear Madam, Dear Sir,

This survey focuses on the topic of **point-of-care ultrasonography (POCUS)**. Completing it should take no more than 10 minutes.

The survey is anonymous, and participation is entirely voluntary. The data collected will be used solely for scientific research purposes.

The survey consists of single-choice and multiple-choice closed questions — please select the answers that best reflect your true opinions. Some questions are open-ended; please respond based on your knowledge and experience. At the end of the survey, a few demographic questions are included.

For a better understanding of point-of-care ultrasonography, below is a definition:
Point-of-care ultrasound (POCUS) is a simplified ultrasound examination performed by a clinician to answer a specific diagnostic question in the context of a patient's clinical symptoms. The POCUS examination typically takes about 3–5 minutes and is currently used across many medical specialties worldwide. Its role and prevalence in daily clinical practice are steadily increasing.

The aim of the study is to evaluate the current level of POCUS utilization in pediatric care in Poland, focusing on pediatricians' self-assessed competencies, perceptions of its clinical utility, and key barriers to its implementation in daily practice.

Thank you very much for completing the survey and for your time!

1. Have you attended any **POCUS** training courses?
Select only one answer.
   - No
   - Just planning
   - Yes

2. How would you rate the availability of **POCUS** training in paediatrics?
Select only one answer.
   - Low
   - Moderate
   - High
   - Sufficient, but the problem is their high cost
   - No opinion

3. Is **POCUS** equipment available at your current workplace?
Select only one answer.
   - No
   - Yes

4. How often do you perform **POCUS** in your daily practice?
Select only one answer.
   - No skills at all
   - Rarely
   - Occacionaly
   - Frequent

5. How do you evaluate your own competence in performing **POCUS**?
Select only one answer.
   - Very low
   - Low
   - Don’t know
   - High
   - Very high

6. How would you rate the current demand for **POCUS** in pediatric practice?
Select only one answer.

- Very low
- Low
- Moderate
- High
- Very high

7. In your opinion, what are the clinical benefits of implementing **POCUS** in everyday pediatric practice?
multiple choice

- Improves diagnostic accuracy
- Reduces time to diagnosis
- Reduces the numer of referrals for more invasive test, e.g. X-ray or CT
- Facilitates clinical decision-making
- Makes work difficult due to time involved
- Bring no benefits

8. Do you think the use of **POCUS** can contribute to increasing the professional prestige of pediatricians?
Select only one answer.

- No impact
- Slight impact
- Don't know
- Major impact
- Significant impact

9. Do you think that acquiring **POCUS** skills and enhancing your professional qualifications would have a positive impact on your job satisfaction?
Select only one answer.

- No
- No opinion
- Yes

10. Do you think that **POCUS** can shorten the diagnostic pathway in pediatric patient?

- No impact
- Slight impact
- Don't know
- Major impact
- Significant impact

11. What additional benefits of **POCUS** performed by pediatricians do you see for doctors, patients, or the healthcare system?

………

………

………

12. Do you believe that using **POCUS** in pediatrics can lead to cost savings in the Polish healthcare system?

Select only one answer.

- No
- No opinion
- Yes

13. What do you think are the main barriers and challenges that hinder the implementation of **POCUS** in pediatric practice within the Polish healthcare system?

multiple choice

- Lack of support from public institutions (e.g., Ministry of Health, NFZ)
- Lack of appropriate training
- Lack of adequate equipment
- Lack of support from hospital management
- Lack of official guidelines from scientific societies

Other: ……………………………………………………………………………………………………

14. What are your suggestions for improving the implementation of POCUS in pediatric practice?

………

………

15. What are the main factors that support the implementation of POCUS in the daily practice?

multiple choice

- Openness of the pediatrician community to innovation and change
- Support from public institutions (e.g., Ministry of Health, National Health Fund)
- Support from scientific societies or organizations involved in postgraduate medical education
- Introduction of mandatory POCUS training in pediatric specialization programs or medical studies

- Support and encouragement from hospital or clinic management staff

  Other: ……………………………………………………………………………………………………

16. Sex
    - Women
    - Men
17. What is your current professional position?
    - Pediatric specialist
    - Pediatric resident
18. How many years of experience do you have in pediatric practice?

    ………
19. Employment sector

    Select all that apply.
    - Inpatient healthcare (hospital setting)
    - Outpatient healthcare
20. Age

    Select only one answer.
    - over 60
    - 45-59 years old
    - 35-44 years old
    - under 34 years old
21. In which voivodeship do you work?

    ………..

This content has not been created or approved by Google Forms
